# Bellevue Hospital

**Published:** 1860-09

**Authors:** 


### BELLEVUE HOSPITAL.

### MALPOSITION OF FŒTUS DETECTED BY EXTERNAL MANIPULATION DURING LABOR—CEPHALIC VERSION BY THE SAME MEANS—SUCCESSFUL.

The following interesting and highly practical questions in obstetrical science, have recently been agitated among those devoted to that branch of medicine:

1. Can a malposition of the fœtus in utero be detected by external manipulation prior to labor?

2. Can such malposition be rectified prior to labor by external manipulation?

Some of the leading professors of obstetrics in this country have taken strong grounds in the negative, and several, not only denying the possibility of detecting and correcting malpositions prior to labor, but even questioning the professional honesty of the practitioner who should endeavor to determine by external examination, prior to labor, whether a malposition existed. The following case, which occurred in Bellevue Hospital, a few days since, under the care of Dr. Barker, is a most important contribution to this subject. We copy the notes of the case, furnished us by Dr. E. B. Barrett, House Physician:

Mary Ann ———, aged 17, born in Ireland, was admitted to the waiting wards of the Bellevue Hospital, March 19th, 1860, in her first pregnancy. On examination, the uterine tumor was very conical, projecting markedly to the right, as well as forwards, triangular in shape, with its transverse diameter greatest. The os uteri was dilated to the size of a quarter of a dollar, and dilatable. The membranes were very full, and projected strongly from the os, and so tense were they that no part of the child could be felt from danger of rupturing them. On the following morning, as the pains had somewhat exhausted the patient, from their teasing character, and as little or no progress was being made, an opiate was administered, from which the patient was enabled to obtain some sleep, awaking much refreshed at 12 M., (the 4th.) The pains now increased in force and frequency, but with no result, save the further dilatation of the os, which was now between two and three inches in diameter. On vaginal examination no part of the child be felt without rupturing the membranes; by palpation, the previous conjecture, viz: that a transverse presentation existed, was rendered nearly certain.

Dr. Barker came to pay his daily visit at this juncture, and concurring in the opinion that the child presented transversely, decided on immediate interference. Accordingly the patient was brought under the influence of chloroform. Dr. Barker again examined the tumor more carefully, both by palpation and per vaginum. Through the external walls the head could be felt in the left iliac fossa, above the pubis. The pelvic extremity of the fœtus was in the right iliac fossa, and the curve of the dorsum could be traced strongly projecting forwards. By a vaginal examination the os was found dilated to the diameter of about three inches, soft and dilatable, with a very large and projecting bag of waters. The presenting part of the fœtus could not easily be felt through the bag of waters, and no force was used for fear of rupturing the membranes; but the deduction, from the external and vaginal examination, was that the right shoulder was the presenting part at the superior strait. Dr. Barker therefore decided to attempt cephalic version in preference to the ordinary method, and he at once proceeded to operate, in the presence of several members of the medical staff of the hospital. The patient being brought to the edge of the bed, in the approved position, he first manipulated by attempting to elevate the pelvis of the fœtus with his left hand, and to depress the head with his right, acting only when it was found that the uterus was not contracting. Finding that he had essentially changed the position of the fœtus, the right hand of an assistant was placed to make strong pressure over the left iliac fossa; he (Dr. B.) still elevating the pelvis of the fœtus with his left, introduced into the vagina two fingers of the right, and during the time of uterine contraction ruptured the membranes, when the waters escaped with great force and abundance. The head could now be felt in the superior strait in the left occipito iliac position. The administration of chloroform was now discontinued, in the hope that the uterus would complete the delivery unassisted. Slight pains, with little progress, followed for fifteen minutes, and, as the fœtal heart was beating somewhat feebly, it was deemed necessary to apply the forceps. The forceps being applied, a male child, weighing eight pounds, was extracted in a partially asphyxiated condition. Respiration was established in a short time, however, and it is now a perfectly healthy child. The placenta came away in five minutes, with no more than the usual amount of hemorrhage; the perineum was not lacerated, and the patient but little more prostrated than is usual after natural labor. The patient proceeded to a rapid convalescence, with no unfavorable symptom, notwithstanding the prevalence of an epidemic of puerperal fever in the Hospital at the time of her confinement.

In his clinical remarks upon the case, Dr. Barker contended that he had demonstrated beyond cavil—

1. That a malposition of the fœtus in utero can be detected by external manipulation during labor. Auscultation furnished but slight assistance, and the results of an internal examination were entirely negative. The important fact, however, worthy of special notice, is, that the position of the fœtus could only be ascertained when the uterus was in a quiescent state. It follows, therefore, if the most favorable time for detecting the position of the fœtus is in the interval between contractions, that a malposition can be made out by external manipulation prior to labor; and if the position can be discovered in the interval of the pains of labor, it can also be done one, or two, or even many days before labor.

2. This case demonstrates the possibility of performing version by external manipulation during labor, and, as version by the hand introduced into the uterus was attempted in the absence of pain, he thought this case equally proved that version by external manipulation is possible before labor. But to have the fœtus remain in its new position it would be necessary to rupture the membranes and induce labor, in order to secure the engagement of the head in the pelvic cavity.

